# r/K‐like trade‐off and voltinism discreteness: The implication to allochronic speciation in the fall webworm, *Hyphantria cunea* complex (Arctiidae)

**DOI:** 10.1002/ece3.3334

**Published:** 2017-11-04

**Authors:** Fan Yang, Eriko Kawabata, Muhammad Tufail, John J. Brown, Makio Takeda

**Affiliations:** ^1^ Graduate School of Agricultural Science Kobe University Nada‐ku Kobe Japan; ^2^ Institute of Tropical Agriculture and Forestry Hainan University Haikou Hainan China; ^3^ Department of Plant Protection College of Food and Agricultural Sciences King Saud University Riyadh Kingdom of Saudi Arabia; ^4^ Department of Entomology Washington State University Pullman WA USA

**Keywords:** allochronic speciation, diapause, genetic divergence, reproductive isolation, voltinism

## Abstract

North America has distinct types of *Hyphantria* moths (Arctiidae) characterized by red (RD)‐ and black (BL)‐headed larvae, of which the taxonomic status is unresolved. Genetic divergence of 26 populations, based on 710 bp of the mtCOI sequence, showed two phylogenetic lineages, which could not be connected in the haplotype network with 95% confidence. The two lineages are separated by 3.1% sequence divergence and should be considered for full species status. The estimated split occurred 1.2–1.6 million years ago. The range of the RD type covered most of the continent, whereas that of the BL type was limited to eastern deciduous forests. Several biological characteristics were differentiated in the zone of cohabitation where BL had more annual generations than RD. Spring emergence of BL precedes that of RD in the field by at least 1 month, because the diapause in BL was shallow, whereas it was deep in RD. Voltinism requires discreteness of numbers, which functions as a sink of hybrids between the two parental lines that have distinct but equally adaptive reproductive strategies; BL may be more r‐strategist‐like and RD more K‐strategist‐like, because fast‐developing BL has multivoltine life cycle, investing less silk proteins as the round‐the‐clock feeder, and slow‐developing RD univoltine one investing more silk as the nocturnal feeder. Also, intensity of diapause, deep in RD and weak in BL, was grossly different, which may enforce segregation of spring adults. Allochronic speciation avoiding coincidental occurrence of adult stages is therefore the most likely scenario. Because the adults never meet in nature, large morphological differentiation is not required.

## 1 INTRODUCTION

The fall webworm, *Hyphantria cunea* (Drury) (Lepidoptera: Arctiidae), originally distributed only in North America, contains at least two distinct types, particularly at the larval stage. Subsequently a sibling species *H. textor* (Harris) was proposed based on spotless adults as the spotless fall webworm, although it was not described formally (Fig. S1). Morris (1963) observed no reproductive barrier between the two forms with different wing patterns, reared larvae of *H. cunea*, and obtained two types of adults from offspring with spotted and spotless wings. He therefore declared *H. textor* a synonym of *cunea*. However, *Hyphantria* contain two types of larvae recognized based on the color of the head capsule and tubercle: a red‐headed type (RD) and a black‐headed type (BL). Their formal taxonomic status is not fixed (Hattori & Ito, 1973; Jaenike & Selander, 1980). BL has invaded and successfully colonized Europe and Asia. Ito and Warren (1973) and Hattori and Ito (1973) reported that the two types interbred to produce viable offspring. However, Gomi, Muraji, and Takeda (2004) investigated two types of *Hyphantria* moths collected both in the Far East Asia and USA, which showed distinct haplotypes of mitochondrial cytochrome c oxidase subunit 1 (COI).

Morris and Fulton (1970) and Ito and Warren (1973) demonstrated that the two types differed in host preference, behavior, and nest structures. They were basically polyphagous, but Oliver (1964) observed that BL preferred sweetgum, persimmon, and willow, whereas RD preferred pecan and persimmon in Louisiana, although we observed BL on a pecan grove in Brownsville‐Harlingen, Texas. BL larvae spin coarse webs and feed all day, whereas RD larvae spin strong webs hiding deep in them during the daytime and feeding mainly at night (Hidaka, 1977; Takeda, 2005). In addition, the proportions of the chemical components in their pheromone blend differed between BL and RD, but the chemical components themselves were identical (Hill, Kovalev, Nikolaeva, & Roelofs, 1982). Field surveys showed that both types are widely distributed throughout Canada, the Gulf coast of Mexico, and the continental USA except Alaska, with large areas of the eastern USA and Canada cohabitated by both types, but the appearance of these types in the field was seasonally segregated due to differences in photoperiodism and diapause parameters (Takeda, 2005).

DNA barcoding, based on a partial sequence of the mitochondrial COI, has been widely used to: characterize intraspecific genetic diversity (Low et al., 2014), identify species status (Hebert et al., 2004), identify the homeland of invasive species (Armstrong & Ball, 2005; Briski, Cristescu, Bailey, & MacIsaac, 2011), and discriminate closely related species and populations (Hebert, Ratnasingham, & de Waard, 2003). In the field of insect molecular systematics, COI sequence variations also provide high reliability and resolution for determining intraspecific and interspecific relationships (Ball & Armstrong, 2006; Xia et al., 2012) and phylogeographical patterns among various insect taxa (Prabhakar et al., 2012). A COI genetic distance threshold is also proposed as a guideline for the delimitation of new species (Lefébure, Douady, Gouy, & Gibert, 2006).

Our investigation of genetic differentiation among *Hyphantria* populations collected in different geographic locations of Japan and North America was designed to determine the species status of highly variable populations of *Hyphantria* moths. Based on the pattern of geographic distribution, behavior, and life cycle patterns, a unique mode of speciation was conjectured.

## 2 MATERIALS AND METHODS

### 2.1 Distributions of red‐headed and black‐headed larvae and adults with unspotted wings and spotted wings

**Figure 1:**
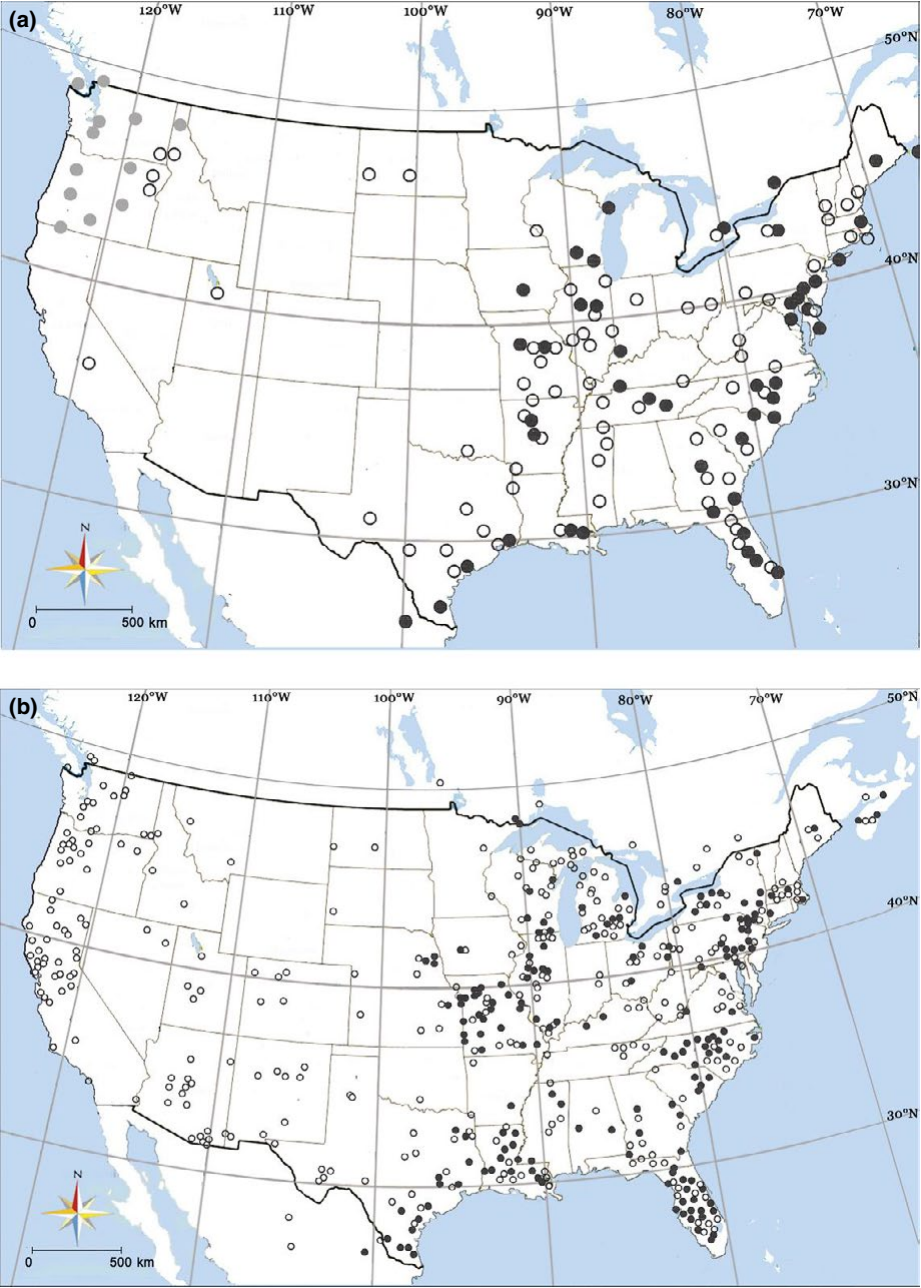
Road survey of larval head capsule color (a) and distribution of wing patterns based on museum specimens (b) of *Hyphantria* moth. (a) ● black‐headed types; ○ red‐headed type; 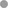 maroon type; (b) ● wings with black spots; ○ spotless wings

The distribution of *Hyphantria* populations in North America in the field was surveyed, and their head color, setae, subcuticular color, and nest types were recorded (Figure 1a). Adult specimens were examined from 37 Institutions (Table S1), and their distributions of wing types, immaculate, and spotted were mapped (Figure 1b).

### 2.2 COI sequencing and analyses of genetic divergence

A total of 198 samples collected from 26 localities between 2006 and 2015: 2 localities in Japan, 1 locality in Canada, and 23 localities in the United States (see Table S2 for detailed sampling information). We identified the type of living larvae upon collection by observing the red (RD) or black (BL) head capsule color, and found a third color type (maroon, MR) in the samples collected from Washington, and Oregon but only west of the Cascade Mountain range. A total of 61 specimens of BL, 98 specimens of RD from both west and east, and 39 specimens from west of the Cascade Mountain range were used in the sequence analysis (Table S1). All individuals were transported with permission from the Plant Quarantine Service, Japan (ex., No. 21‐Kobe‐616). Specimens used in this study were either dried pinned specimens, fresh, or preserved in absolute ethanol and stored at −80°C.

Prior to DNA extraction, all the specimens were washed in 80% ethanol. Total genomic DNA was extracted and purified from larvae, pupae, and adults using a GenElute (TM) Mammalian Genomic DNA Miniprep Kit (Sigma, St Louis, MO) in accordance with the manufacturer's protocols. The primers used to amplify 710 bp of the COI region were C1‐J‐1718 5′‐GGAGGATTTGGAAATTGATTAGTTCC‐3′ and TL2‐N‐3014 5′‐TCCAATGC ACTAATCTGCCATATTA‐3′ (Gomi et al., 2004). The amplification program was as follows: An initial step of 5 min at 95°C followed by 35 cycles of 30 s at 94°C, 30 s at 50°C, and 1 min at 68°C, which was followed by a final extension of 10 min at 68°C. Each reaction was conducted in a total volume of 50 μl containing 30 ng genomic DNA, 10 μl of 2 mM dNTPs mix, 25 μl of PCR buffer, 1 μl of KOD FX Neo polymerase (Toyobo, Osaka, Japan), and 5 μl of each 10 μM forward and reverse primer sets. Sequencing was performed using an ABI 3130xl DNA sequencer with a Big Dye Terminator Kit ver3.1. To exclude possible PCR errors, unique haplotypes among all samples were confirmed by additional amplification and sequencing. All of the haplotypes obtained in this study have been submitted to the DDBJ under accession numbers from LC136937 to LC136970 (Table S3).

Five sequences from the United States that we deposited previously in DDBJ/GenBank were retrieved (AB675111, AB675486, AB675487, AB675488, and AB675489). A multiple alignment of all the sequences was conducted using ClustalW and edited manually in BioEdit (Hall, 1999). Based on these aligned sequences, determination of haplotypes and construction of statistical parsimony haplotype network were carried out using TCS 1.21 (Clement, Posada, & Crandall, 2000). This program was designed to calculate the maximum number of mutational differences between haplotypes with the probability of 90% according to the algorithm described in Templeton, Crandall, and Sing (1992). It is also used extensively to differentiate species and to infer genealogies at the population level for low levels of divergence (Gómez‐Zurita, Petitpierre, & Juan, 2000; Hart & Sunday, 2007). Pairwise genetic distances were determined using MEGA v. 5.0 (Tamura et al., 2011) for the Kimura 2‐parameter (K2P) model that best fit our data according to the test performed in jModeltest v.0.1.1 (Posada, 2008). To visualize the distances, the neighbor‐joining (NJ) tree was presented using MEGA v. 5.0.

Genetic diversity of two types of populations was quantified by assessing haplotype diversity, nucleotide diversity in the program DnaSP 5.0 (Librado & Rozas, 2009). Tajima's D (Tajima, 1989) and Fu's *F*s (Fu, 1997) were examined for deviation from neutrality, as an indicator of past population expansion. Significant negative values of D and *F*s were expected as being characteristic of population expansion. An analysis of mismatch distribution was applied to calculate the frequency distribution of the number of differences between pairs of sequences. A unimodal approximate Poisson‐like distribution is expected for populations that have experienced population expansion in the recent past, whereas multimodal frequency distribution is expected for demographic equilibrium (Rogers & Harpending, 1992).

### 2.3 Phenology, diapause intensity, and adult size

The phenology of local populations was reconstructed based on data from museum specimens and local moth catch records. Cumulative % emergence on specific calendar dates was converted to probits by looking up those corresponding to the percentage responded in Finney's table (Finney, 1952).

To compare diapause intensity between BL and RD, mature larvae were collected from the field in 1982, fall and brought in the laboratory and feeding was continued in the laboratory. RD are all from New Haven, CT but collection of BL larvae is difficult and only one nest was found in New Haven, two nests from New York City (BL population), and Washington, D.C. (BL population) were used to supplement the experiment. Diapause pupae were divided into groups (*N* = 30–35) upon pupation when they were exposed to a temperature of 5°C in constant darkness (DD). The diapause pupae of RD were chilled at 5°C for 20, 60, 90, 120, and 260 days, while 125, 200, and 260 days for BL populations. After chilling treatment for the designated period, diapause pupae were incubated at 25°C LD 16:8, and the number of days until 50% of individuals became adults was recorded.

Additionally, diapause intensity was examined nation‐wide. Diapause intensity changes during cold storage, which is expressed as the number of days till 50% of adults emerged after variable periods of chilling at 5°C. Fall generation field‐collected larvae of two types were kept in the laboratory and reared until pupation. The period of chilling treatment was 17, 31, 47, 68, 82, 96, 110, 130, 151, 165, and 179 days for BL populations collected in Baton Rouge, Louisiana; 154 and 206 days for RD‐E (east coast) collected in Woodville, Mississippi; 29, 97, 178, 206, and 224 days for RD‐W (red‐headed and maroon‐headed from west coast) collected in Sand Point, Idaho and 123, 137, and 157 days for RD‐W (west coast) collected in Pullman, Washington. Each group (20–30 pupae) was treated for a different period without mixing. Curve fitting adjustments and the logistic regression were performed with MATLAB when the relationship of two variables was not linear. Mann–Whitney *U*‐test was used to determine whether significant differences occurred in diapause intensities among BL, RD‐E (east coast), RD‐W (west coast) populations.

To compare their morphological characteristics between populations showing sympatry and allopatry, adults specimens of BL, RD‐E, and RD‐W were measured for the forewing length using a micrometer. The comparison was made in two ways: first using adults grown in the laboratory on the same artificial diet in the last instar and second using field‐collected pinned specimens stored in the museum. The second comparison was made for east and west at almost the same latitudinal zone. Size can be compared between different voltinism zones. All data were analyzed by analysis of variance with the Tukey–Kramer multiple comparisons tests.

## 3 RESULTS

### 3.1 Distributions of larvae with distinct head capsule color and adults with different wing patterns

The survey map showed that BL larvae spun a loose web (Fig. S1e), and they occurred only in the eastern USA and Canada down through the Gulf side of Mexico, whereas RD larvae were more widely distributed both in the east and west (Figure 1a). The latter type of larvae spun denser webs (Fig. S1f). The two types of larvae can be distinguished by coat color also (Fig. S1c,d), but an intermediate type of head and coat color has never been encountered in the area of cohabitation. In the Northwestern USA, MR larvae can be observed, particularly to the west of the Cascade Mountains, but a completely black head color was never observed in the field in the west. The nest type (Fig. S1g) was also distinct from the nest of the BL type. The distribution of adult specimens with spotted wings (Figure 1b, filled circle; Fig. S1a) closely matched with the distribution of BL larvae (Figure 1a, filled circle; Fig. S1c).

### 3.2 Genetic divergence of *Hyphantria* populations

**Figure 2:**
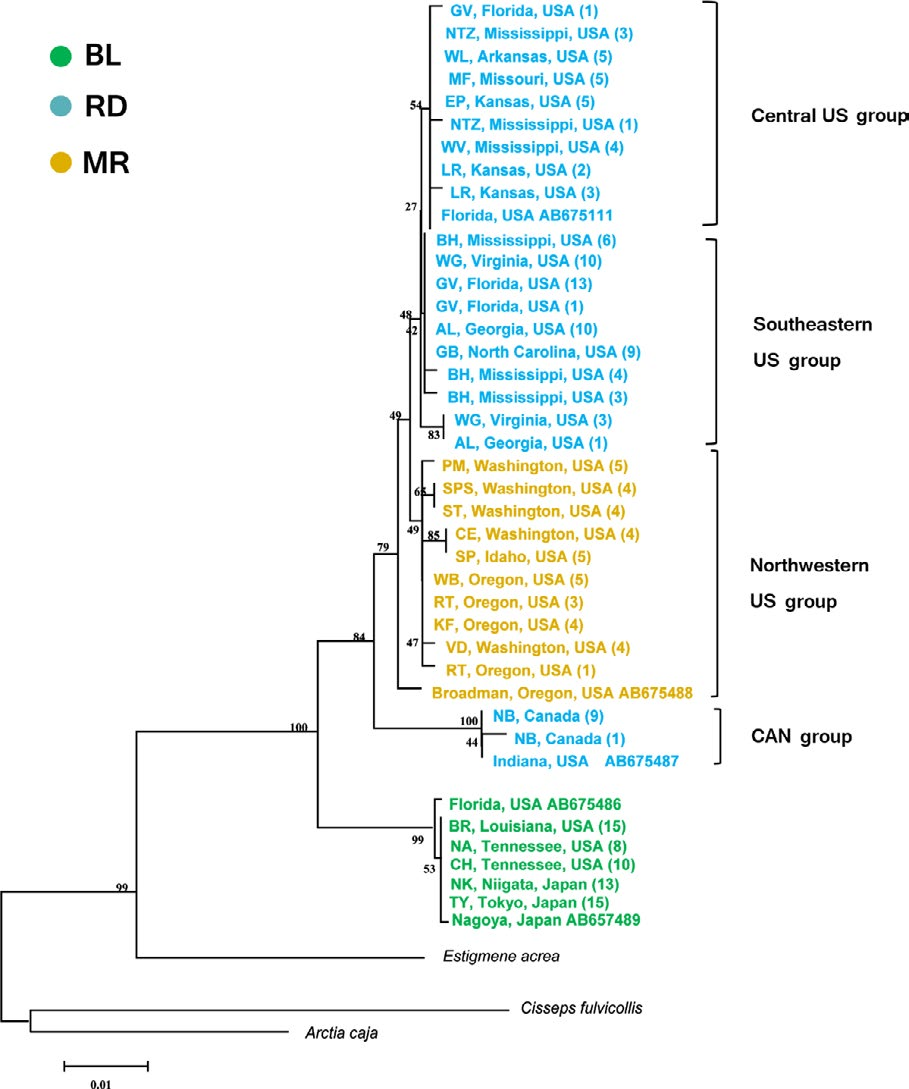
Phylogeny of *Hyphantria* populations reconstructed using a neighbor‐joining tree based on Kimura‐2‐parameter distances with a confidence level assessed using 1,000 bootstrap replications. A total of 203 COI sequences (198 from this study and 5 from DDBJ/GenBank) were included

Figure 2 demonstrates that 26 geographic populations were clustered into two major branches with high bootstrap values, corresponding, respectively, to BL and RD. Northwestern USA populations including MR were in the RD cluster. The 203 aligned COI sequences were 710 bp long (198 sequenced in this study plus 5 from GenBank), and all alignments were unambiguous and no insertions nor deletions were detected.

**Figure 3:**
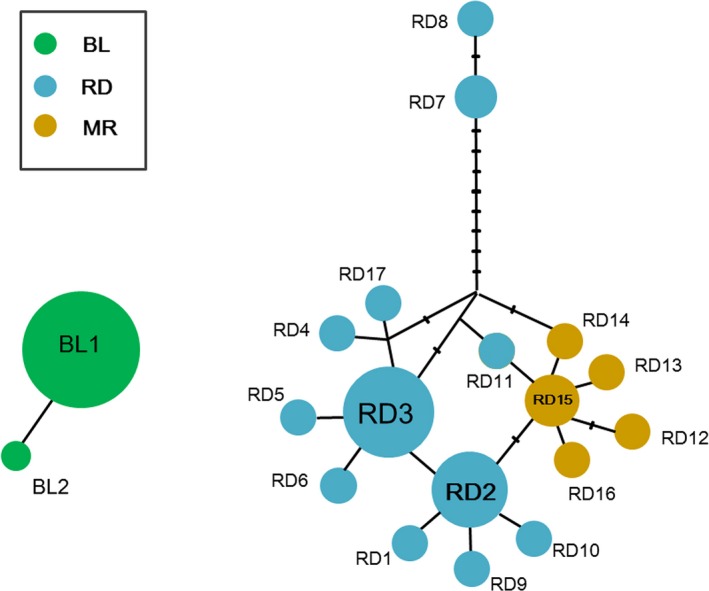
Relationship among haplotypes of the 710‐bp COI sequence of *Hyphantria* populations presented in a parsimonious joining network. The size of the circle indicates the number of individuals sharing the particular haplotype. Connecting haplotypes specify the number of mutation steps separating haplotypes. Haplotype code and locality details are shown in Table S1 and Table 1

Two unconnected networks in TCS showed two haplotypes in BL populations (BL1 and BL2) and 17 haplotypes in the RD populations (RD1–RD7; Figure 3). In the RD network, three star‐like clades, with three haplotypes (RD2, RD3, and RD15) occupying the center, were detected, which corresponded to geographic clusters in the phylogenetic reconstruction (Central, Southeastern, and Northwestern USA Groups). Two haplotypes, RD7 and RD8, originating from Canada were the most genetically distant from the other haplotypes.

### 3.3 Genetic structure and historical demography

**Figure 4:**
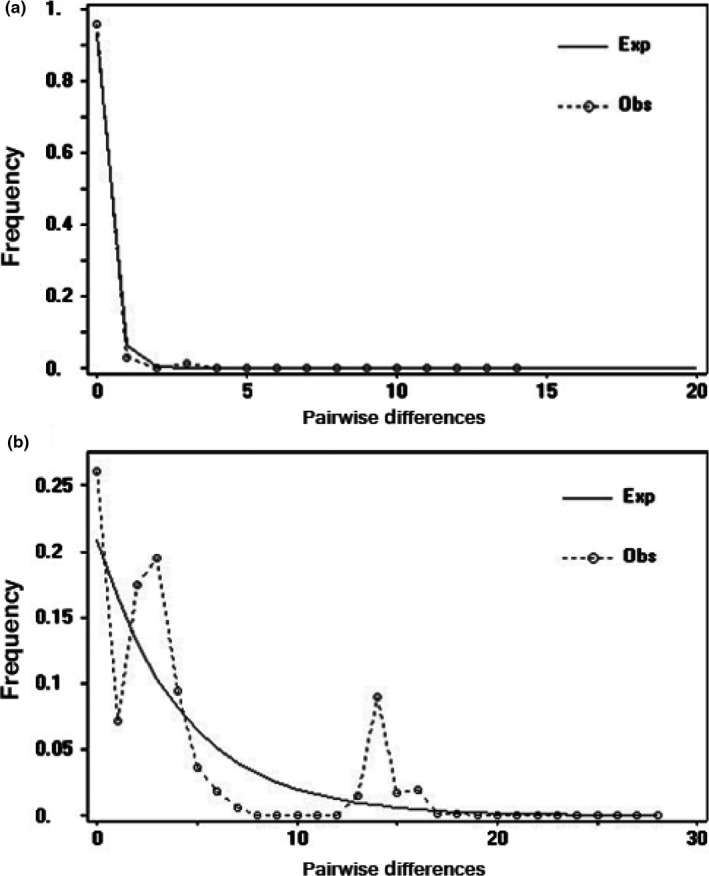
Mismatch distribution of BL (a) and RD populations (b). The solid line is the theoretically expected mismatch distribution, and the dotted line is the observed mismatch distribution

The RD populations showed higher nucleotide and haplotype diversity (h = 0.00533, pi = 0.740 ± 0.039) than the BL populations, whose haplotype and nucleotide diversity values were 0.00004 and 0.032 ± 0.022, respectively (Table 1). The value of Tajima's D and Fu's *F*s was negative for BL populations and significantly deviated from neutrality across the entire data set (*F*s = −4.27406, *p* < .01). The mismatch distribution of BL populations was distinctly unimodal (Figure 4a), which suggested that they had experienced natural selection or expansion (Table 1). In the RD populations, both tests for population expansion using Fu's *F*s (*F*s = −1.357, *p *> .05) and bimodal mismatch distribution analysis (Figure 4b) suggested that this population was in equilibrium.

**Table 1:** Molecular indices calculated for the BL and RD populations based on COI sequence in *Hyphantria* moths

Index	BL populations	RD populations
Polymorphic sites	2	41
Nucleotide diversity (pi)	0.00004	0.00533
Haplotype diversity (h)	0.002 ± 0.022	0.740 ± 0.039
Tajima's *D*	−1.86622*	−0.85844^ns^
Fu's *F*s	−4.27406**	−1.357^ns^
Genetic distance range	0.000–0.001	0.001–0.023

ns, not significant.

*p < .05; **p < .01.

### 3.4 Seasonal phenology of different geographic groups

**Figure 5:**
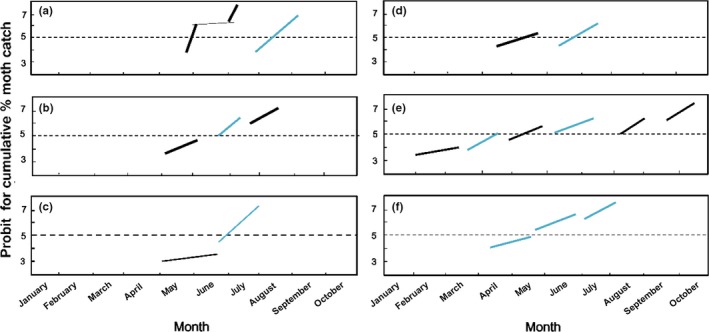
Observation of moth flight across the season (probit‐transformed accumulated percentage). Two types of wing forms were plotted separately: Black lines represent those with spotted wing, and blue lines represent spotless adults. (a) Slovakia (left) and New Brunswick, Canada (right); (b) Missouri (Takeda, 2005); (c) Geneva, NY; (d) Combined data from New York, Pennsylvania, Wisconsin, Maine, Massachusetts, and New Jersey; (e) Mississippi, Georgia, Florida, and Tennessee combined; (f) Oregon and Washington combined

The two types of adults, with spotted wings and spotless wings (Fig. S1a,b), emerged at different times. Our laboratory rearing of 40 years showed that RD larvae always produced spotless adults, whereas BL larvae produced both spotless and spotted adults, depending on the rearing temperatures and photoperiod. Moth catch based on collection dates of museum specimens and light trap data were used to identify voltinism and seasonal phenologies in various regions (Figure 5). Figure 5a shows probit‐transformed cumulative % adult emergence in Slovakia (left, modified from Jasič, 1964) and New Brunswick, Canada (modified from Morris & Fulton, 1970). In Slovakia, despite apparent bimodality, only BL is known as univoltine. In New Brunswick, most likely only one generation occurs based on Morris and Fulton (1970) (Figure 5a right). Takeda (2005) presented the seasonal life cycle patterns of fall webworms in mid‐central Missouri based on light trap data and museum specimens cross checked by field observation (Figure 5b). In this area, two generations of BL and one generation of RD were observed in the field (2BL‐1RD zone). Field observation showed bivoltinism with BL and univoltinism with RD, and the two types never overlapped in the field at the same developmental stage except diapausing pupae in winter. Figure 5c is based on the light trap catch at the New York Agricultural Experiment Station at Geneva (courtesy of the late Dr. Ada Hill). The major peak appeared in June (most likely RD), but small number of adults that did not belong to the major peak population preceded the major peak. These adults may represent the BL populations, but a second BL peak was missing at this latitude (1BL‐1RD zone), unlike in Missouri. It is important to know whether such seasonal isolation of reproductive stage occurs in all areas, including the northern areas (1BL–1RD zone) and the southern areas that produce multiple BL and RD generations. Therefore, seasonal phenology was reconstructed from compiled data for collection dates in the Northern USA above 40°N (Figure 5d). The results confirmed the seasonal segregation in adult flight between these two wing types. Adult emergence of these types may overlap in the Southern USA in a multivoltine area where both RD and BL undergo more than two generations a year. Figure 5e shows compiled flight data for southern populations from the latitude zone below 31°N. The results clearly showed that four generations of BL and two generations of RD were seasonally segregated except in late March. Two peaks partially overlapped but the earlier portion of the spotless peak may be BL, because BL produces spotless adults during the summer. For comparison, Northwestern USA populations were plotted in the same way (Figure 5f). This suggested a mixed life cycle showing both univoltinism and bivoltinism but flight occurs more or less continuously, a mode unlike the East Coast USA sympatric populations of RD. The seasonal pattern in moth flight in Oregon and Washington was more gradual, and flight peaks were not so clearly separated as in the Eastern USA sympatric populations.

### 3.5 Diapause intensity determines the appearance of the first generation

**Figure 6:**
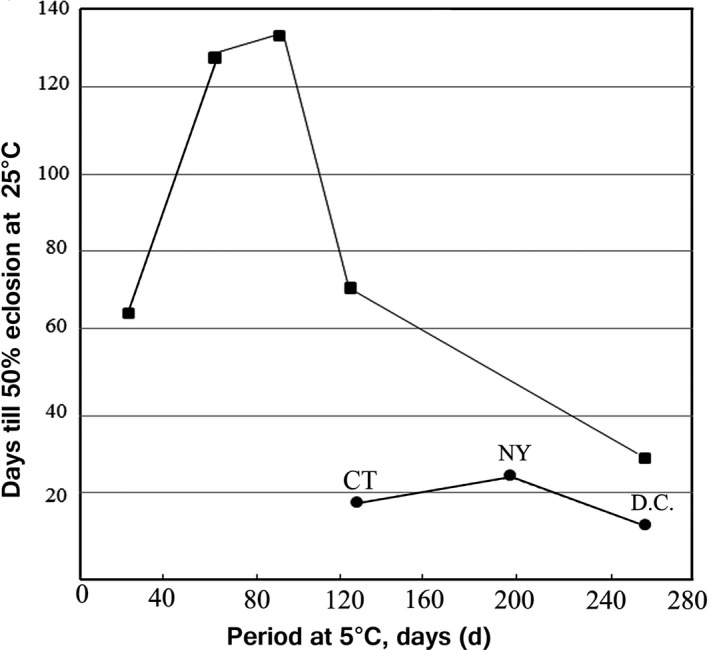
Changes in diapause intensity. Days to 50% pupal eclosion at 25°C LD 16:8 after a designated period of chilling, DD. RD (■) and BL (•) larvae were collected in fall, 1982 in New Haven (CT) and BL (•) collected in New Haven (CT), New York City (NY) and Washington, D.C. (D.C.). *N* > 30 in each treatment

**Figure 7:**
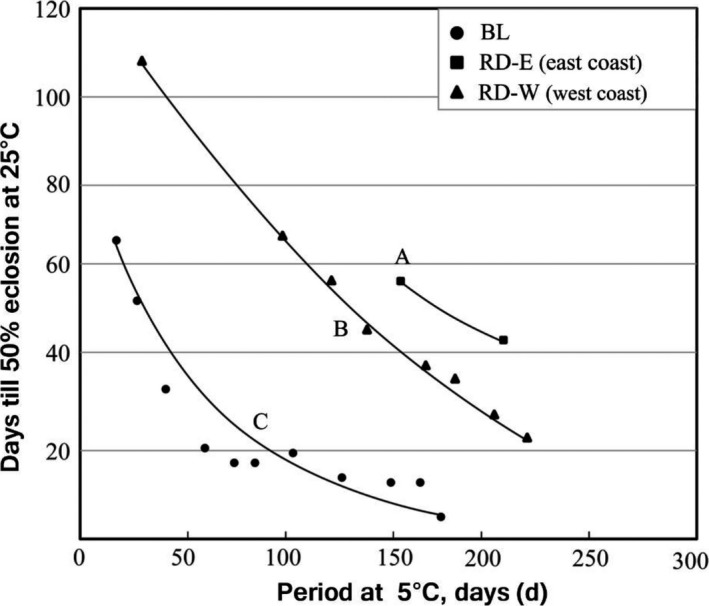
Evidence for character displacement in diapause intensity. Different alphabets (A, B and C) indicate significant differences by the Mann–Whitney *U*‐test with Bonferroni's correction, *p *< .01. BL populations collected from Baton Rouge, LA; RD‐E populations collected from Woodville, MS; RD‐W populations collected from Pullman, WA and Sandpoint, ID

Figure 6 illustrates a striking difference in diapause intensity between the BL and RD from the same voltinism zone. Diapause had already ended after 4 months of chilling (see also Figure 7 showing that diapause of BL requires about 50 days of chilling for 50% individuals to terminate it). In contrast, diapause of the RD was intensified initially by chilling, and even after 90 days of chilling, 4 months were required for 50% of individuals to emerge.

Figure 7 compares the intensity of diapause among the three groups, BL, RD‐E, and RD‐W. Overall, the results showed that both the RD‐E and RD‐W had deeper diapause than BL. After only 50 days of chilling, the intensity of diapause in BL reached almost the lowest level, from which about 20 days more were required for adult differentiation and morphogenesis, whereas about 140 days of chilling was required to terminate diapause in 50% of RD individuals. The difference at 90 days should be significantly great in terms of producing the first moth flight in the field. It should be noted that intensity of diapause differed between the two geographical groups of RD, namely RD‐E and RD‐W. Diapause of RD‐E was deeper than that of RD‐W, although populations used were from more southerly location than Oregon‐Washington in spite that with multivoltinism option toward southern territories, the intensity of diapause was expected to be the reverse of the latitudinal cline, that is, deeper diapause in the northern population than in the southern population.

### 3.6 Comparison of body size and diapause intensity among BL, RD‐E and RD‐W

The body size of West Coast USA RD (RD‐W) was significantly larger than those in Eastern USA RD (RD‐E) and BL populations (Table 2).

**Table 2:** Comparison of adult body size (mm) among two types of *Hyphantria* moths. Values shown are means ± *SE*. Means in each column followed by different letters are significantly different by one‐way ANOVA followed by Tukey–Kramer multiple comparisons tests

	Type	Sex	*N*	Maximal length of forewing
Laboratory‐reared specimen^a^	BL	Female	18	25.7 ± 1.19c
	RD‐E		15	26.8 ± 2.31b
	RD‐W		20	29.7 ± 1.12a
	BL	Male	18	11.8 ± 1.24b
	RD‐E		21	12.3 ± 1.03b
	RD‐W		13	14.6 ± 1.56a
Field specimen				
In the south^b^	BL	Male	25	14.4 ± 0.89b
	RD‐SE		17	14.6 ± 1.05b^d^
	RD‐SW		21	15.8 ± 1.17a
In the north^c^	BL	Male	20	14.4 ± 0.91b
	RD‐NE		19	14.2 ± 1.01b^d^
	RD‐NW		25	16.2 ± 1.06a

a The BL specimens were collected in Baton Rouge, LA; RD‐E (east coast) specimens were collected in Woodville, MS; RD‐W (west coast) specimens were collected in Pullman, WA.

b The BL specimens (south east) and RD‐SE (south east) were collected in NC and SC; RD‐SW (south west) specimens were collected in AZ.

c The BL specimens (north west) and RD‐NE (north west) were collected in NY, MA and Canada; RD‐NW (north west) specimens were collected in OR.

d No significant difference was detected between RD‐SE and RD‐NE by Student's *t*‐test.

## 4 DISCUSSION

### 4.1 Several clades in *Hyphantria* moths and the species status

Previously, differentiation in morphology, pheromone blend, behavior, and seasonal life cycle has been examined between the two types of *Hyphantria*, and no easily observable break point had been found between them. However, a critical breakpoint for the demarcation of species should not always be sought in external morphology. Here, we first investigated genetic differentiation mainly based on mitochondrial COI sequences. Molecular phylogenetic analysis of *Hyphantria* populations collected from 26 localities (Japan, Canada, and United States) resulted in two major clades represented by BL and RD. MR was nearly identical to RD and, therefore, belongs genetically to the RD clade. The average K2P distance between the BL and RD (plus MR) (3.1%) was slightly greater than the interspecific threshold of 3% observed for Lepidoptera by Hebert, Cywinska, and Ball (2003). Our previous studies also showed that the level of divergence between the two types was 3.5%–4.0% for the *COIII* gene, and 2.7%–3.5% for *cyt b* gene (Gomi et al., 2004). Two types of wing pattern are produced from BL larvae, which are probably seasonal morphs. RD larvae always produce spotless winged adults. This probably belongs to another species. Their lifecycle patterns also showed a difference in adult appearance time in the field (Figure 5). BL has a weak diapause, emerging in spring but RD emerges much later due to a deeper diapause (Figure 6). This gives an effective mechanism for reproductive isolation. The distribution, morphology, life cycle, and mitochondrial data all agreed that the genus *Hyphantria* has two species: BL and RD.

Jaenike and Selander (1980) investigated eight enzyme‐coding loci and also concluded that two types were completely differentiated and that they deserved full species status. The present COI data supported and extended this notion. Additionally, we partially cloned a circadian clock gene, *period*, and found that RD and BL had distinct alleles and that there was no evidence of cross‐over among populations belonging to each species, indicating that the two species did not hybridize with each other, that is, speciation was complete (unpublished data).

Congruence between larval morphology and wing patterns showed that the BL and RD types were both sympatrically and allopatrically distributed in North America (Figure 1). The RD type was widely distributed across North America, but the BL type was restricted to the eastern United States, Canada, and Mexico, and the MR type was restricted to the Northwestern USA, particularly to the west of the Cascade Mountains. The RD (plus MR) populations displayed an obvious phylogeographic structure, high genetic variation, and a more constant population size indicated by the multimodal distribution, together strongly implying the nature of genetic signature and long‐term stable distribution. Conversely, the significant Fu's *F*s value and unimodal distribution indicated historical instability in the BL populations. The low level of genetic diversity in BL was perhaps related to population expansion after amelioration of climatic or biotic stresses. The BL population rapidly expanded its territory to the west but failed to penetrate the western territory of maternal RD species, probably because it preferred plants adapted to the more humid climate prevailing in eastern North America. However, without fossil records and age identification of the ecological background, reconstruction of colonization history is not possible within North America.

### 4.2 Allochronic speciation?

The substitution rate for insect mitochondrial DNA was estimated to be 2.3% per million years (Brower, 1994). Using this rate, the divergence of BL and RD dates back to about 1.2–1.6 Ma. Although the origin of the split based on COI sequence corresponds to the late Pleistocene events (Calabrian), it is often difficult to distinguish clearly between historical events and other deterministic evolutionary factors, such as continental split or climate change or glacial cycles, which could give rise to the observed patterns of genetic differentiation. To the best of our knowledge, there was no geographic gap in the Cenozoic era. Both sides of the Appalachian Mountain are currently inhibited by both BL and RD. The Rocky Mountains and the Mississippi River hypothesis have been examined by Masaki and Ito (1977), but the effectiveness of these barriers must be limited because the RD populations are currently found in the Rocky Mountains area along the river or creak lines, and the area splitting the distribution was near the tornado corridor no matter how wide the Mississippi River was at that time.

Alternative modes of speciation (i.e., sympatric speciation) have been presented (Johnson & Gullberg, 1998; Kondrashov, Yampolsky, & Shabalina, 1998; Maynard Smith, 1966). Kaneko and Yomo (2000) demonstrated that sympatric speciation is theoretically possible, when two distinct phenotypes keep a symbiotic relationship. It is a premise that sufficiently strong nonrandom mating and spatially heterogeneous selection are required in a model for sympatric speciation (Gavrilets, 2006). A common and well‐documented mode of sympatric speciation involves host plant shift, such as seen in *Rhagoletis* flies (Bush, 1969). However, *Hyphantria* is polyphagous, and it was also found that there was a weak relationship between host plants and genetic differentiation (Loewy, 2013), which does not support host race formation by sympatry.

The allochronic speciation hypothesis was first presented by Alexander and Bigelow (1960) for the North American *Gryllus* species. *G. pennsylvanicus and G. veletis*, which overwinter at egg and nymphal stages, respectively. This split in overwintering stage inevitably splits the reproductive season between the two types, through which speciation was realized, according to the authors. This hypothesis was, however, refuted by Harrison (1979) based on allozyme analysis, and later on mitochondria DNA (Harrison & Bogdanowicz, 1995) analyses showing remote lineages of these species. The case of *Rhagoletis* flies (Bush, 1975) implicates host race formation as a primary cause, but difference in timing of fruiting in two potential hosts, apple and hawthorn, leading to a split in mating time is also a critical factor. The original Alexander‐Bigelow hypothesis collapsed, but recently in two cases, it was supported by genetic data (Santos, Paiva, Rocha, Kerdelhué, & Branco, 2013; Santos et al., 2011; Yamamoto & Sota, 2009, 2012). These cases dealt with univoltine species and a shift in diapause stage easily caused segregation of two forms with different life cycle patterns. However, essentially polyphagous *Hyphantria* moths have potentially multivoltine capacities, and the mechanism to maintain the two types requires more sophisticated tuning.

Observation for sympatric populations in central Missouri suggested that differentiation in developmental rate and critical photoperiod resulted in the separation of two species, favoring univoltinism for the RD species and bivoltinism for the BL species (Takeda, 2005). Univoltinism and bivoltinism may provide a bistability condition in association with lifecycle and anti‐predatory strategies. Oliver (1964) reported the timing of emergence of overwintering generation of the black phenotype, that is, BL species here, was about 1 month earlier than that of the red phenotype, that is, RD species here, in Louisiana, which was consistent with our analysis (Figure 2e). Hybridization between univoltine paternal stock and bivoltine maternal stock, for example, may produce 1.5 generation a year, which would be fatal, because voltinism must be discrete in number to survive a stressful season at the same development stage resistant to stress. Although a theoretical possibility exists that sporadic hybridization may occur, hybrids cannot survive and have never been observed in the wild, and the moth flight record showed discrete peaks in the Northern, Central, and Southern USA for both species (Figure 5). The adult emergence times of the overwintering generation are seasonally segregated by differentiation in diapause intensity (Figures 6 and 7), and allochronic speciation provides most likely explanation.

### 4.3 Interspecific competition

Initial congeneric competition may have resulted in sympatric speciation allochronically that reduced competition (Dieckmann & Doebeli, 1999). Competition is reflected both in BL and RD‐E in the eastern territories in some biological characteristics, whereas RD‐W in the west had no competitive rival, providing a neutral condition in this aspect (Figure 7, Table 2). The body size of RD‐W was significantly larger than those of RD‐E and BL (Table 2). This could be a footprint of past competition between sympatric populations. Competition must cut short in resources and time to grow. RD‐W may enjoy more seasonal resources and developmental time, whereas RD‐E and BL types have more confluent life cycle due to the presence of competitors. Avoidance of this competitive season should be more beneficial for both species; it can be facilitated by differentiation in photoperiodism (Takeda, 2005) and in diapause intensity; deep diapause in RD‐E and shallow in BL (Figure 7), which results in mutual exclusion in seasonal adult emergence time. Seasonal segregation should expose the worm to different ecological conditions in resource quality and abundance, parasite and predator complex, and climatic conditions. This competition could cause character displacement in many biological characteristics between RD and BL types (Table 3).

**Table 3:** Biological traits where differentiation between the two types of *Hyphantria* moths was noticed in previous investigations

Trait	BL	RD	References
Larval head color	Black	Orange/Red‐dark brown	Oliver (1964)
Coat color	White‐black	Light brown‐black	Ito and Warren (1973)
Adult wing pattern	Spotted and immaculate	Immaculate	Present study
Nest	Weak^a^	Strong^b^	Ito and Warren (1973)
Distribution	East coast of North America	Whole North America	Present study
Feeding rhythm	Round‐the‐clock^a^	Nocturnal^b^	Takeda (2005)
Number of annual generation	1–4 generation per year^a^	1–2 generation per year^b^	Present study
Diapause	Shallow^a^	Deep^b^	Present study
Rate of development	Fast^a^	Slow^b^	Takeda (2005)
Host plant preference	Mulberry, willow, persimmon	Walnut, pecan, sycamore	Ito and Warren (1973) and Oliver (1964)

a r‐strategy.

b K‐strategy.

### 4.4 r/k selection

Any new phenotype must be sustained in the ecological background. Seasonal segregation in mating time is an effective mechanism for assortative mating for distinct ecological adaptations. Allochronic speciation could occur in *Hyphantria* more efficiently if it was supported by a demographic strategy. Seasonal niches exert differential r/K selection. Bivoltinism conforms an r‐like strategy, that is facilitated by fast development, comparatively less spinning, and the round‐the‐clock feeding. Univoltinism results from intensive investment for silk spinning, nocturnal feeding that slow development, conforming a K‐like strategy (MacArthur & Wilson, 2015; Parry, 1981). In sympatric populations in Missouri, BL has shallower diapause producing the first generation earlier in the season than RD, consuming resources rapidly and constructing less protective nest. They are round‐the‐clock feeders and have a shorter larval period than RD, inevitably exposing themselves to natural enemies, that are day active (Takeda, 2005). RD heavily invest in nest materials, hiding themselves in the nest core during the day time, but thereby sacrificing feeding time and the number of generations. This represents two sets of ecological option in a trade‐off. If both ecological strategies equally merit and are sustained stably in the temporal structure of ecosystem, once the common gene pool splits and the split populations may be stably maintained thereafter (bistability). Natural selection should be against hybridization that produces a nondiscrete number of generation. This mode of ecological accommodation might have occurred in East Coast USA where the BL genotype was generated.

## CONFLICT OF INTEREST

None declared.

## AUTHOR CONTRIBUTIONS

Makio Takeda contributed to the conception of the study. Fan Yang, Eriko Kawabata, and Makio Takeda contributed significantly to analysis and manuscript preparation; Fan Yang and Makio Takeda performed the data analyses and wrote the manuscript; Makio Takeda and John J. Brown collected live materials and field data in the US; Fan Yang and Makio Takeda helped perform the analysis with constructive discussions.
