# Supplementary figures and images for: r/K‐like trade‐off and voltinism discreteness: The implication to allochronic speciation in the fall webworm, Hyphantria cunea complex (Arctiidae)
Source: Ecol Evol. 2017 Nov 4;7(24):10592–603. doi: 10.1002/ece3.3334 (PMC5743571; doi:10.1002/ece3.3334)

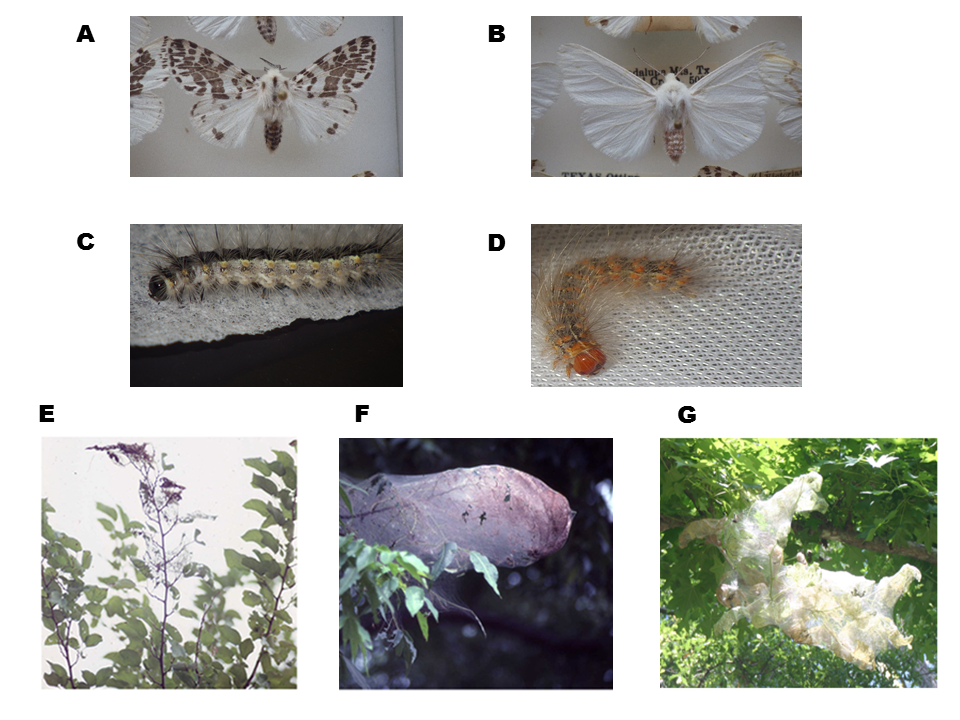

Supplement: Supplementary file 1 [file ECE3-7-10592-s001.tif]
