# Supplementary material for: r/K‐like trade‐off and voltinism discreteness: The implication to allochronic speciation in the fall webworm, Hyphantria cunea complex (Arctiidae)
Source: Ecol Evol. 2017 Nov 4;7(24):10592–603. doi: 10.1002/ece3.3334 (PMC5743571; doi:10.1002/ece3.3334)
Supplement: Supplementary file 2 [file ECE3-7-10592-s002.docx]

**Supporting Information**

**r/K bistability and voltinism discreteness; a possible cause of allochronic speciation in the fall webworm,** ***Hyphantria cunea* complex (Arctiidae)**

Fan Yang^1^, Eriko Kawabata^1^, Muhammad Tufail ^1, 2^, John J. Brown^3^, Makio Takeda^1^*

1 Graduate School of Agricultural Science, Kobe University, Rokko-dai 1-1, Nada, Kobe ,657-8501, Japan

2 Department of Plant Protection, College of Food and Agricultural Sciences, King Saud University, Riyadh, P.O. Box 2460 Riyadh 11451, Kingdom of Saudi Arabia.

3 Department of Entomology, Washington State University, Pullman, WA USA

* Corresponding author: Makio Takeda, E-mail: [mtakeda@kobe-u.ac.jp](file:///D:\日本学习资料\28s%20and%20co1%20paper\manuscript\%20mtakeda@kobe-u.ac.jp)

Tel: +81-78-803-5870

Fax: +81-78-803-5870

Table S1. List of Institutions where specimens were examined.

| University | Univ. of Missouri, Univ. of Florida, Univ. of Michigan, Univ. of Minnesota, Univ. of Tennessee, Univ. of Kentucky, Univ. of Maryland, Univ. of Massachusetts, Univ. of Delaware, Univ. of Georgia, Purdue Univ., Ohio State Univ., Mississippi State Univ., Oklahoma State Univ., Kansas State University, North Carolina State Univ., Louisiana State Univ., Arizona State Univ., Washington State Univ., Oregon State Univ., Michigan State Univ., Univ. of Arkansas, Harvard Univ., Cornell Univ., Clemson Univ., Univ. of California at Berkeley, Iowa State Univ., New Mexico State Univ., Texas A&M Univ., Colorado State Univ. |
| --- | --- |
| Institute | Smithsonian Institute, Virginia Polytech. Inst |
| Museum | American Museum, San Francisco City Museum, Fields Museum at Chicago and Canadian Forestry Service at New Brunswick |

.

Table S2. Details of *H. cunea* sampling sites. BL, black-headed type; RD, red-headed type; MR, maroon-headed type.

| **ID** | **Country** | **Origin** | **Head color** | **No.** | **Latitude** | **Longitude** | **Date of collection** |
| --- | --- | --- | --- | --- | --- | --- | --- |
| BR | United States | Baton Rouge, LA | BL | 15 | 30°28'02.0"N | 91°09'50.4"W | Aug.2008 |
| NA | United States | Nashville, TN | BL | 8 | 36°12'10.0"N | 86°48'30.0"W | Aug.1997 |
| CH | United States | Chattanooga, TN | BL | 10 | 35°42'14.0"N | 85°18'16.0"W | Aug.1997 |
| NK | Japan | Nagaoka, Niigata | BL | 13 | 37°26'23.7"N | 138°51'37.6"E | Aug.2014 |
| TY | Japan | Toyosu, Tokyo | BL | 15 | 35°39'10.5"N | 139°47'45.5"E | Aug.2015 |
| NB | Canada | Fredericton, New Brunswick | RD | 10 | 45°31'87.2"N | 66°39'54.3"W | Oct. 2015 |
| BH | United States | Brookhaven, MS | RD | 13 | 31°51'32.7"N | 90°24'03.4"W | Oct. 2015 |
| WG | United States | Woodbridge, VA | RD | 12 | 38°39'18.8"N | 77°15'03.0"W | Oct. 2015 |
| GV | United States | Gainesville, FL | RD | 15 | 29°27'15.9"N | 82°18'17.1"W | Oct. 2015 |
| AL | United States | Atlanta, GA | RD | 11 | 33°22'19.5"N | 84°49'42.0"W | Oct. 2015 |
| GB | United States | Greensboro, NC | RD | 9 | 35°48'31.0"N | 80°10'51.0"W | Oct. 2015 |
| MF | United States | Marshfield, MO | RD | 5 | 37°20'16.2"N | 92°55'48.6"W | Sept. 2007 |
| EP | United States | Emporia, KS | RD | 5 | 38°23'43.2"N | 96°14'30.9"W | Sept. 2007 |
| LR | United States | Lawrence, KS | RD | 5 | 38°59'22.9"N | 95°13'05.4"W | Sept. 2007 |
| WL | United States | Winslow, ARK | RD | 5 | 35°48′10.5"N | 110°25'37.9"W | Sept. 2007 |
| NTZ | United States | Natchez, MS | RD | 4 | 31°34′17.1"N | 91°19'13.3"W | Sept. 2007 |
| WV | United States | Woodville, MS | RD | 4 | 30°48′26.1"N | 94°25'22.3"W | Sept. 2007 |
| PM | United States | Pullman, WA | RD | 5 | 46°44′52.2"N | 117°13'28.6"W | Aug.2008 |
| CE | United States | Cle Elum, WA | MR^a^ | 4 | 47°11′08.5"N | 120°57'31.5"W | Aug.2008 |
| SP | United States | Sandpoint, ID | MR^a^ | 5 | 48°16′56.9"N | 116°33'12.3"W | Aug.2008 |
| VD | United States | Vader, WA | MR | 4 | 46°22′47.1"N | 122°56'22.0"W | Aug.2008 |
| ST | United States | Seattle, WA | MR | 4 | 47°37′51.0"N | 122°19'23.1"W | Aug.2008 |
| SPS | United States | Snoqualmie Pass, WA | MR^a^ | 4 | 47°24′39.0"N | 121°24'30.2"W | Aug.2008 |
| WB | United States | Woodburn, OR | MR | 5 | 45°09′10.3"N | 122°49'52.1"W | Aug.2008 |
| KF | United States | Klamath Falls, OR | MR^a^ | 4 | 42°13′34.4"N | 121°47'30.6"W | Aug.2008 |
| RT | United States | Ritter, OR | RD | 4 | 44°54′14.3"N | 119°08'33.1"W | Aug.2008 |

^a^ Not determined

| Population ID | Haplotype | GenBank accession no. |
| --- | --- | --- |
| BR | BL1(15) | LC136937 |
| NA | BL1(8) | LC136968 |
| CH | BL1(10) | LC136967 |
| NB | RD7(9),RD8(1) | LC136965, LC136966 |
| BH | RD3(6),RD5(4),RD6(3) | LC136943- LC136945 |
| WG | RD3(10),RD4(3) | LC136969, LC136970 |
| GV | RD1(1),RD3(13),RD2(1) | LC136938-LC136940 |
| AL | RD3(10),RD4(1) | LC136941, LC136942 |
| GB | RD3(9) | LC136946 |
| MF | RD2(5) | LC136947 |
| EP | RD2(5) | LC136948 |
| LR | RD2(2),RD10(3) | LC136949, LC136950 |
| WL | RD2(5) | LC136951 |
| NTZ | RD2(3),RD9(1), | LC136952, LC136953 |
| WV | RD2(4) | LC136954 |
| PM | RD11(5) | LC136955 |
| CE | RD12(4) | LC136956 |
| SP | RD12(5) | LC136957 |
| VD | RD13(4) | LC136958 |
| ST | RD14(4) | LC136959 |
| SPS | RD14(4) | LC136960 |
| WB | RD15(4) | LC136961 |
| KF | RD15(4) | LC136962 |
| RT | RD15(3),RD16(1) | LC136963, LC136964 |

Table S3. Haplotypes of COI identified in each population.


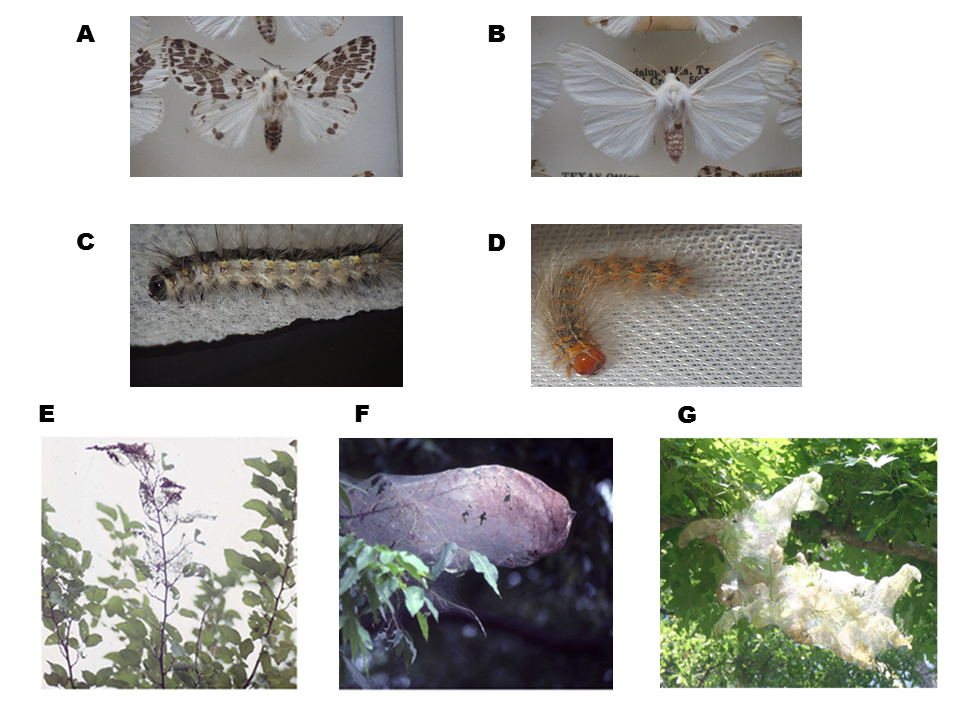


Figure S1. Adult wing pattern, larval coat color and nest structure. (A) Spotted wing; (B) Immaculate wing; (C) BL with black coat color; (D) RD with red coat color; (E) Coarse webs spun by BL larvae; (F) Strong webs spun by RD larvae; (G) Denser webs spun by MR larvae in the West of Cascade Mountain.
